# Generation of Transgenic Cloned Buffalo Embryos Harboring the EGFP Gene in the Y Chromosome Using CRISPR/Cas9-Mediated Targeted Integration

**DOI:** 10.3389/fvets.2020.00199

**Published:** 2020-04-23

**Authors:** Xiuling Zhao, Junyu Nie, Yuyan Tang, Wengtan He, Kai Xiao, Chunying Pang, Xianwei Liang, Yangqing Lu, Ming Zhang

**Affiliations:** ^1^State Key Laboratory for Conservation and Utilization of Subtropical Agro-Bioresources, Animal Reproduction Institute, Guangxi University, Nanning, China; ^2^Key Laboratory of Buffalo Genetics, Breeding and Reproduction Technology, Ministry of Agriculture and Buffalo Research Institute, Chinese Academy of Agricultural Science, Nanning, China

**Keywords:** sex control, CRISPR/Cas9, HDR, Y chromosome, p53, PFT-μ

## Abstract

Sex control technology is of great significance in the production of domestic animals, especially for rapidly breeding water buffalo (bubalus bubalis), which served as a research model in the present study. We have confirmed that a fluorescence protein integrated into the Y chromosome is fit for sexing pre-implantation embryos in the mouse. Firstly, we optimized the efficiency of targeted integration of exogenous gene encoding enhanced green fluorescent protein (eGFP) and mCherry in Neuro-2a cells, mouse embryonic stem cells, mouse embryonic cells (NIH3T3), buffalo fetal fibroblast (BFF) cells. The results showed that a homology arm length of 800 bp on both sides of the target is more efficient that 300 bp or 300 bp/800 bp. Homology-directed repair (HDR)-mediated knock-in in BFF cells was also significantly improved when cells were supplemented with pifithrin-μ, which is a small molecule that inhibits the binding of p53 to mitochondria. Three pulses at 250 V resulted in the most efficient electroporation in BFF cells and 1.5 μg/mL puromycin was found to be the optimal concentration for screening. Moreover, Y-Chr-eGFP transgenic BFF cells and cloned buffalo embryos were successfully generated using CRISPR/Cas9-mediated gene editing combined with the somatic cell nuclear transfer (SCNT) technique. At passage numbers 6–8, the growth rate and cell proliferation rate were significantly lower in Y-Chr-eGFP transgenic than in non-transgenic BFF cells; the expression levels of the methylation-related genes *DNMT1* and *DNMT3a* were similar; however, the expression levels of the acetylation-related genes *HDAC1, HDAC2*, and *HDAC3* were significantly higher (p < 0.05) in Y-Chr-eGFP transgenic BFF cells compared with non-transgenic cells. Y-Chr-eGFP transgenic BFFs were used as donors for SCNT, the results showed that eGFP reporter is suitable for the visualization of the sex of embryos. The blastocyst rates of cloned buffalo embryos were similar; however, the cleavage rates of transgenic cloned embryos were significantly lower compared with control. In summary, we optimized the protocol for generating transgenic BFF cells and successfully generated Y-Chr-eGFP transgenic embryos using these cells as donors.

## Introduction

Producing offspring with the desired sex is a significant goal in livestock production. Several approaches have been used to determine the sex of embryos prior to embryo transfer in mammals. Histocompatibility-Y antigen, which serves as a male-specific antigen, has been used to identify the gender of bovine embryos (1). Non-radioactive *in situ* hybridization (2) and fluorescence *in situ* hybridization (3) using a labeled Y-specific probe have been performed to determine the sex of human and bovine embryos. Sex chromosome-based PCR analysis has also been used to determine the sex of embryos (4–6). However, these methods are generally error prone, labor intensive and can even be detrimental to normal embryonic development (7, 8). Therefore, it is of interest to develop non-invasive methods to determine the sex of pre-implantation embryos. One successful example is the production a male mouse line with a labeled X chromosome (9).

It is also of interest to develop methods to improve the efficiency of generating transgenic animals, especially livestock. Genetically modified livestock are produced to satisfy the need for food or non-food products (10–12). However, whereas genetically modified mice can be easily obtained through genetic modification of embryonic stem cells (ESCs) or direct embryo injection, isolating and culturing ESCs for livestock is difficult and pronuclear microinjection is less efficient (13, 14). Therefore, the most popular method for producing genetically modified livestock is somatic cell nuclear transfer (SCNT) (15–17). The major advantage of SCNT over direct embryo injection is the predictable genotype of the offspring and the ability to generate clonal lines of edited animals (18). A variety of transgenic livestock models have been produced with improved growth rate, resistance to disease, and more efficient feed usage using somatic cell cloning techniques (19–21).

Unlike embryos derived from fertilization, most cloned embryos die before and after implantation, and even those that can survive to term are frequently defective, vulnerable to miscarriage, high birth weight or perinatal death (22–26). DNA methylation, histone methylation and histone acetylation are closely related to the developmental potential of cloned embryo in mammals (27, 28). Histone acetylation is an important event in epigenetics, and reprogramming of histone acetylation in donor cells is very important for turning the nucleus of a highly differentiated state into a pluripotent state. HDACs are histone deacetylases, hypoacetylated histones are related to lower transcriptional activity due to closed chromatin structure formation (29). There are considerable evidences to suggest that the abnormal epigenetic reprogramming of cloned embryos is an important cause for its low development potential (30–32). Studies have shown that the DNA methylation status of donor cells can also affect the efficiency of nuclear reprogramming, and the level of DNA methylation is inversely related to the developmental potential of cloned embryos (33, 34). DNMT1 and DNMT3a are DNA methyltransferases (35), responsible for the maintenance of DNA methylation and the establishment of *de novo* methylation during early embryonic development. The lower the DNA methylation level of the donor cell, the higher the embryonic development ability (36–39), and reducing the DNA methylation level of donor cells can improve the developmental efficiency of cloned embryos (36, 40–42). However, some studies have found that 5-aza-2'deoxycytidine (5-aza-dC), a DNA demethylation drug, cannot effectively improve the development efficiency of cloned embryos (43–46). This result indicates that the relationship between the DNA methylation status of donor cells and the development efficiency of cloned embryos needs to be further clarified. The efficiency with which transgenic embryos are created using SCNT is mainly determined by the ability to obtain genetically modified somatic cells. Many types of somatic animal cells can be used as donor cells for SCNT (47–49). Fetal fibroblasts have become widely used to produce transgenic livestock for several advantages. For example, they are easy to obtain and culture, and can be genetically modified and develop into live offspring. However, the low efficiency of introduction and integration of exogenous DNA into the genomes of primary mammalian cells, such as fetal fibroblast cells, has greatly limited the production of transgenic animals by SCNT. Other factors that may affect the efficiency of transgenic animal production efficiency are DNA methylation and histone acetylation, which are involved in remodeling the genome and are linked to gene imprinting in the early embryo, and cell viability.

One major method used to integrate exogenous DNA into the genome is homology-directed repair (HDR), nevertheless, the efficiency of HDR is very low (<1%) without selection (50–52). To overcome this limitations, researchers have employed the type II bacterial clustered regularly interspaced short palindromic repeats (CRISPR)/Cas9 system, which has been engineered into an efficient genome-editing tool consisting of the Cas9 nuclease and a single guide RNA (sgRNA) (53, 54). At present, CRISPR/Cas9 mediated HDR is the commonly used approach to achieve precise and targeted integration of transgenes because of its high efficiency. There are some reports that the length of the homology arm (HA) can dramatically influence the efficiency of targeted integration of exogenous genes (55, 56). Therefore, it may be possible to further improve the efficiency of CRISPR/Cas9-mediated HDR in the cells by optimizing HA length.

The integration of exogenous DNA into genome is based on DNA damage and repair by the CRISPR/Cas9 system. Severe deleterious consequences of Cas9-induced DNA damage, mainly DNA double-strand breaks (DSBs), have been observed in some studies (57, 58). What's more, some researchers have found that a single targeted DNA break is enough to cause cell cycle arrest or apoptosis in some cell types (59, 60). Further experimental evidence in human pluripotent stem cells showed that introduction of DSBs at a single locus is sufficient to cause a P53-dependent molecular response and that transient suppression of the activity of p53 can improve the efficiency of precise genome engineering (60). Transient suppression of p53 activity can be achieved by treating cells with Pifithrin-μ (PFT-μ), a small molecule that inhibits binding of p53 to mitochondria by reducing its affinity to the anti-apoptotic proteins Bcl-xL and Bcl-2 but has no effect on p53-dependent transactivation (61).

Guangxi province has the largest number of buffalo in China, and is the main force driving the development of China's dairy buffalo industry. Increasing the number and improving the quality of dairy herds are crucial ways to accelerate the development of Guangxi's dairy buffalo industry. Here, we used buffalo as a research model for optimizing two factors that limit the genetic modification of somatic cells: the introduction of exogenous DNA and the integration of the DNA into the genome. Specifically, we optimized the parameters for introduction of exogenous DNA into buffalo fetal fibroblast (BFF) cells using electroporation, which is one of the most widely used methods for introducing exogenous DNA into somatic cells because of its high efficiency. We also optimized methods to improve the efficiency of exogenous gene integration in BFF cells using the CRISPR/Cas9 system. Our previous research revealed that a labeled Y chromosome is suitable for the identification of the sex of pre-implantation mouse embryos (62). In this study, we developed a Y chromosome-linked eGFP mouse line that stably expresses green fluorescent protein under the control of the CAG promoter. A tracer to the Y chromosome has simplified the process of predicting the sex of embryos collected by mating a Y-Chr-eGFP transgenic male with a wild-type female, all XY embryos appeared green, under a fluorescence microscope, and XX embryos did not. The accuracy of this method can reach 100%, which provides an expeditious and accurate approach for sexing pre-implantation embryos. To confirmed the applicability of this method in livestock, buffalo served as a model. We successfully established procedures for producing transgenic cloned buffalo embryos with the *eGFP* gene integrated at a specific locus on the Y chromosome using the CRISPR/Cas9 system combined with SCNT. We then compared Y-Chr-eGFP transgenic cells and non-transgenic BFFs in terms of various parameters related to cell growth and proliferation, and expression level of epigenetic-related genes. Finally, we compared the developmental competence of embryos produced by cloning using Y-Chr-eGFP transgenic and non-transgenic BFFs as donor cells.

## Materials and Methods

All animal experimental protocols were performed in accordance with the relevant ethical guidelines and regulations. Buffalo ovaries used for the *in vitro* production of mature oocytes used as SCNT recipients were collected from a slaughterhouse in a suburban area near Nanning City, China.

Unless otherwise stated, all organic and inorganic reagents were purchased from Sigma-Aldrich Co. (St. Louis, MO, USA). Self-made solutions were filtered through a 0.22-μm filter (Millipore, Bedford, MA, USA) and stored at 4°C or at −20°C until use. Pipette tips, centrifuge tubes and petri dishes were purchased in aseptic packages and were all disposable.

### Construction of the CRISPR/Cas9 Plasmid and the Homologous Recombination Repair Vector

Design and construction of the CRISPR/Cas9 plasmid were performed based on the method described by Ran et al. (63). An sgRNA targeting intron 12 of Ddx3y (Gene ID: 783057) was designed using the CRISPR design tool (http://tools.genome-engineering.org). Plasmid px459 (Addgene, catalog no. 62988) was linearized by digestion with the BbsI restriction enzyme (NEB catalog no. R0539S) and ligated with the annealed sgRNA using T4 DNA ligase (Thermo Fisher Scientific, catalog no. EL0011). DNA sequencing was performed to confirm the accuracy of the fully constructed CRISPR/Cas9 plasmid.

To construct the homologous recombination (HR) donor, CAG-EGFP, the 5′ and 3′ HA sequences were amplified from CAG-GFP-IRES-CRE (Addgene, catalog no. 48201) or the animal genome, then subcloned into the donor vector by restriction enzyme digestion and ligation.

### The Design and Screening of sgRNAs

The RFP-GFP-surrogate (RGS) reporter system was used for the screening of sgRNAs (64). This system consists of a red fluorescent protein (RFP) and the 20 bp target sequence plus a 3-bp PAM sequence and a GFP cassette. However, the DNA sequence of GFP is out of reading frame, the reading frame of GFP gene may be recovered by repair of DNA damage when the target sequence ahead of GFP gene is cut. The RGS plasmid was linearized by EcoRI and BamHI. Then we inserted the sgRNA with the PAM into the linearized plasmid. The RGS and px459 plasmid carrying the sgRNA were cotransfected into HEK 293T cells. The transfected cells expressed RFP, and the cleavage rate of sgRNA was measured by determining the percentage of cells expressing GFP.

### Cell Culture and Transfection

Mouse ESCs were cultured in 2i medium, which consists of Dulbecco's modified Eagle's Medium (DMEM) (Gibco), supplemented with 10% fetal bovine serum (FBS), 1,000 U/mL mouse Lif, 2 mM glutamine (Sigma), 1% penicillin/streptomycin (Thermo Fisher Scientific), 0.1 mM non-essential amino acids (Gibco), 1 μM PD0325901 and 3 μM CHIR99021. Mouse Neuro-2a (N2a) and 3T3 cells were cultured in DMEM/F12 (Gibco) containing 10% FBS, 1% penicillin/streptomycin (Solarbio), and 0.1 mM non-essential amino acids (Gibco).

All cells were cultured at 37 °C in a humid atmosphere of 5% CO_2_. Transfection of the cells (mES, N2a, and NIH3T3) was performed using Lipofectamine 3000 Reagent (Invitrogen) according to the manufacturer's instructions. Plasmids containing sgRNA, Cas9 and a donor were transfected into the cells, and 48 h later, transfection-positive cells were sorted by flow cytometry for further analysis.

### Preparation of Buffalo Fetal Fibroblasts

Buffalo fetuses aged 3–4 months were taken from a local slaughterhouse and transported back to the laboratory within 2 h. The amniotic membrane of the fetus was removed with tweezers, and the fetus was washed two to three times in phosphate-buffered saline (PBS) containing penicillin and streptomycin. After washing, the skin tissue was cut into small pieces with sterilized ophthalmic surgical scissors, then placed in a culture plate with a small amount of culture medium and kept in a cell incubator. After culturing for 72 h, the tissue blocks were removed and the cells were continuously cultured for another 2 days, when the adherent cells approached confluence, then stored in liquid nitrogen.

### Optimizing Voltage and Pulse Number to Maximize Delivery Efficiency of Exogenous DNA Into Buffalo Fetal Fibroblast Cells

To examine the effect of voltage on the efficiency of plasmid delivery to fibroblast cells, 20 μg purified vector DNA was mixed into 100 μl PBS containing 1.5 × 10^6^ cells. A single pulse of 0, 100, 150, 200, 250, or 300 V for 1 ms was administered to the BFF cells. Following electroporation, the cells were resuspended in 3 ml of culture media and seeded on a 60-mm culture dish. Thirty-six hours after transfection, each well was washed with PBS and fresh medium containing Hoechst 33258 was added. Plates were then incubated for 20 min at 37°C. The total number of cells in each well was counted based on Hoechst nuclear staining visualized under a fluorescence microscope, and the ratio of the cells expressing eGFP in a field of view (the counts from more than five fields of view were averaged) was determined. Survival rate was defined as the total number of cells divided by the number of cells in the 0 V group in one field of view (an average of five fields were observed per well).

Based on the results of a single pulse at multiple voltages, 250 V was chosen to evaluate the effect of multiple pulses. BFF cells were subjected to 1, 2, 3, 4, or 5 pulses (1 ms each) of 250 V, and 36 h after electroporation, electro-transfection efficiency was analyzed.

### Transfection and Selection of Gene-edited Cell Colonies

Two days before electroporation, the frozen cells were thawed and seeded in a 60-mm cell culture dish (Axygen), and transfection was performed only when confluence reached 70–85%. For electroporation to introduce DNA into somatic cells, three 1-ms pulses of 250 V were applied to 100 μl of 2 × 10^6^ cells in the presence of 10 μg px459 vector and 10 μg donor plasmid. For electroporation, square wave pulses were administered through a BTX ECM 2001 in 2-mm gap cuvettes. Thirty-six hours later, the media was changed to introduce 1.5 μg/mL puromycin (Solarbio, Beijing, China). Cells were incubated for 4 days, and individual cell colonies formed after withdrawing puromycin were continuously cultured for an additional 7–10 days. Individual colonies expressing the GFP genes under fluorescence microscope were picked up and cultured in a 96-well cell culture plate. When confluence was achieved, the cell colonies were sub-cultured to obtain cells for genotyping and SCNT.

### The Genotyping Analysis of the Cells Expressing GFP Genes

Single cell clones expressing green fluorescence were expanded and cultured for genotyping, and primers were designed to the identify the accuracy of integration junction sites. 5′ and 3′ junction sites of exogenous gene were amplified by PCR, the primers used for amplification of 5′ and 3′ junctions were listed in Table S3, then sequenced the PCR products of clones which were double positive at 5′ and 3′ junctions. Choosing the clones that were precise integrations as nuclear transfer donor cells.

### The Expression Level of Epigenetic Related Genes

The expression level of epigenetic related genes was analyzed by real-time quantitative PCR. Total RNA was isolated from BFF and Y-Chr-eGFP transgenic BFF cells using the TRIzol reagent (Invitrogen, USA) according to the manufacturer's instruction. The cDNA was obtained from ~1 μg RNA and reverse transcribed by the PrimeScriptTM RT Reagent Kit with gDNA Eraser (Takara, Japan). Real-time quantitative PCR were performed using CFX96 C1000 Thermal Cycler (Bio-Rad, USA) by TB Green® Premix Ex Taq™ (Tli RNaseH Plus) (Takara, Japan) in triplicate. PCR was performed using two steps for 40 cycles at 95°C for 5 s, 60°C for 30 s and followed by melt curve. All the gene expression levels were normalized to the internal standard gene Gapdh. Relative gene expression was determined using 2^−ΔΔ*Ct*^ method. The primer sequences are listed in Table S4.

### Cell Cycle and MTT Assay

BFF cells were incubated in the presence of PFT-μ for additional 24 and 48 h, subsequently, PI and RNase A were added the collected cells for 30 min of incubation at 37°C. Cells were analyzed by flow cytometry (BD Biosciences) and data analysis was performed using Flow Jo 10.

Cell viability was assessed using the MTT Cell Proliferation and Cytotoxicity Assay Kit (Beyotime, Shanghai, China). Briefly, BFF cells were digested and seeded into 96-well plates at a density of 2 × 10^3^ cells per well. Next, cells were incubated with MTT solution for 4 h at 37°C. Formazan solution was subsequently added to the 96-well plates to dissolve the formazan crystals that formed. The optical density (OD) was measured at 570 nm.

### Production of Y-Chr-eGFP Buffalo Embryos via SCNT

SCNT was performed as described previously (65–68). Briefly, gene-edited fibroblasts were thawed and cultured in serum starvation conditions (DMEM/F12 supplemented with 0.5% FBS) for 2 days to synchronize the cell cycle. The cells were harvested and resuspended with micromanipulation medium (10 mM HEPES-buffered TCM-199 containing 0.3% [w/v] bovine serum albumin; pH = 7.3). Cumulus-oocyte complexes were aspirated from the follicles and cultured in preheated maturation medium (bicarbonate-buffered TCM-199 supplemented with 5% estrous bovine serum and 10 μg/mL follicle-stimulating hormone [FSH]). The oocytes were cultured in an incubator at 39°C under 5% CO_2_ for another 22–24 h. Oocytes with an easily defined first polar body and homogenous cytoplasm were selected for use as nuclear transfer recipients.

Matured oocytes were enucleated by aspirating the first polar body plus a portion of the adjacent cytoplasm, then the donor cell nucleus was injected into the perivitelline space. The fusion of nuclear-transferred embryos was performed using the following program: two successive DC pulses of 2.1 kV/cm for 30 μs under a BTX ECM 2001 electro cell manipulator. Three hours after the fusion, the activation of reconstructed embryos was induced by exposure to 5 μM ionomycin in embryo culture medium for 5 min and subsequent incubation in 2 mM 6-dimethylaminopurine for 4 h at 39°C and 5% CO_2_.

After activation, reconstructed embryos were placed in IVC medium (modified Tyrode's medium supplemented with 36% TCM-199, 10% FBS, 0.06 mg/mL penicillin and 0.1 mg/ml streptomycin) in a 20-μl culture droplet overlaid with mineral oil under a humidified atmosphere of 5% CO_2_ at 39°C. The granulosa cell monolayers were established at least 12 h before the introduction of embryos. The culture medium was refreshed every 48 h after the introduction of embryos by replacing half of the original medium with a similar volume of fresh medium.

### The Development of Cloned Embryos From Y-Chr-eGFP Transgenic BFF Cells

Embryonic cleavage rate and blastocyst formation rate were two parameters we monitored during *in vitro* embryonic development. The blastocyst rate was recorded on day 8 of IVC. Cleavage rate and blastocyst formation rate between BFF cells and Y-Chr-eGFP transgenic BFF cells were analyzed on experiments repeated five times using one-way ANOVA.

### Statistical Analysis

The data are presented as the mean±SEM. At least three replicates were tested for each group. Statistical analyses were performed using Prism 7 software (Graph Pad, San Diego, CA, USA), using analysis of variance (ANOVA) and Student's *t*-tests where appropriate.

## Results

### Preparation of Buffalo Fetal Fibroblast Cells and Optimization of the Concentration of Puromycin

**Figure 1:**
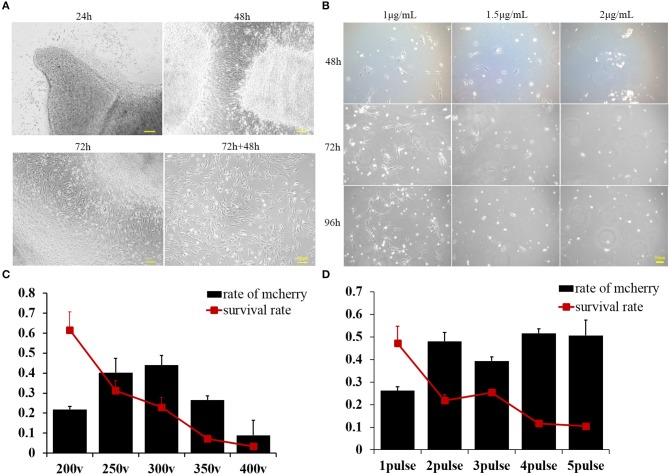
**(A)** Buffalo fetal fibroblast cells were isolated and cultured using the explant technique for 72 h, then continuously cultured for another 2 days after removing the minced tissues. Pictures were taken at 24, 48, and 72 h after culturing and again 48 h after removing the minced tissues. **(B)** Buffalo fetal fibroblast cells were cultured in the presence of different concentrations of puromycin, and pictures were taken after 48, 72, and 96 h. **(C)** The percentage of transfected cells as determined by mCherry expression was affected by voltage. Cell survival was also greatly affected by the voltage. **(D)** The percentage of cells effectively transfected with supercoiled vector and expressing mCherry, was affected by the number of pulses. Cell survival was also greatly affected by the pulse number. Scale bar = 50 μm.

BFF cells were derived from buffalo fetal skin using the direct adherent culture method. Tissue pieces were seeded in a culture dish, and fibroblasts derived from the minced tissues were observed with increasing incubation time (Figure 1A). Seventy-two hours later, tissue pieces were removed from the dishes and cultured for another 2 days until the fibroblasts reached 70–80% confluence (Figure 1A). Cells were then frozen in liquid nitrogen until further use. To optimize the concentration of puromycin, the culture medium was supplemented with increasing doses of puromycin (1, 1.5, and 2 μg/mL). Almost all of the BFF cells treated with 1.5 μg/mL puromycin for 4 days died (Figure 1B). Therefore, 1.5 μg/mL was determined to be the minimum concentration that can be used for screening BFFs carrying puromycin resistant gene.

### Optimization of the Voltage and Pulse Number Used to Deliver Exogenous DNA Into Buffalo Fetal Fibroblasts

A plasmid containing a mCherry reporter gene with expression driven by the CAG promoter was used to estimate the cell transfection efficiency. The percentage of transfected cells was determined by monitoring the expression of mCherry in the cytoplasm of cells. We concluded that voltage can greatly affect the transfection efficiency, with higher voltages producing more transfected cells; however, cell survival decreased with increasing voltage delivered as a single pulse (Figure 1C). Given that 250 V maximized both transfection efficiency and cell survival, this voltage was used to further optimize the number of pulses. According to our second experiment, we deemed that two pulses can achieve the maximum delivery of exogenous supercoiled vector DNA into BFFs without compromising survival (Figure 1D).

### The Length of the Homology Arm Affects the Efficiency of CRISPR/Cas9-mediated Homologous Recombination

**Figure 2:**
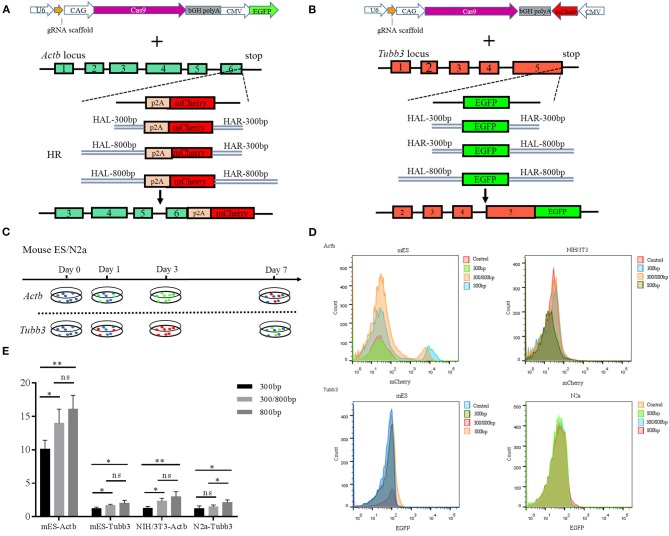
**(A)** Schematic overview of the strategy used to target the *Actb* locus. HAL/HAR, left/right homology arm; HR, homologous recombination; **(B)** Schematic overview of the strategy used to target the *Tubb3* locus; **(C)** Experimental scheme for targeted knock-in of *Actb-p2A-mCherry* in mouse ES and NIH/3T3 cells and *Tubb3-EGFP* in mouse ES and N2a cells. Cells were transfected with donors/GFP or donors/mCherry and Cas9/sgRNA/mCherry/GFP, and transfected cells were sorted based on GFP or mCherry signals 2 days after transfection. **(D)** Knock-in efficiencies were evaluated by fluorescence activated cell sorting (FACS) and were based on the ratio of GFP^+^ or mCherry^+^ cells to all transfected cells; **(E)** Histograms show relative knock-in efficiencies for different homology arm lengths used for knock-in at *Actb* and *Tubb3* in mouse cells expressed as the percentage of mCherry^+^ (or GFP^+^) cells among all transfected cells. Results were presented as mean ± s.d. *P < 0.05, **P < 0.01, ns, no significant difference, unpaired Student's *t*-test.

We next determined whether the length of the HA affects the knock-in efficiency when using an HR-based method. We constructed three donor plasmids with HAs of 300 bp on both sides of the target, 300 bp on one side and 800 bp on the other, and 800 bp on both sides. To assess the knock-in efficiencies, we aimed to fuse a *p2A-mCherry* gene to the last codon of the *Actb* gene in the mouse cell lines ES and NIH3T3, and an *eGFP* reporter gene to the last codon of the *Tubb3* gene in the mouse cell lines ES and N2a (Figures 2A,B). The experimental scheme for targeted *Actb-p2A-mCherry* and *Tubb3-EGFP* knock-in in mouse ES, NIH3T3, and N2a cells is shown in Figure 2C. Seven days after transfection, the knock-in efficiencies for HAs of different lengths were evaluated by flow cytometry and are presented as the percentage of cells expressing the specific reporter protein (Figures 2D,E). We found the highest knock-in efficiency when using an HA of 800 bp for all three types of cell lines.

### PFT-μ Can Improve the Efficiency of Exogenous Gene Integration Mediated by CRISPR/Cas9

**Figure 3:**
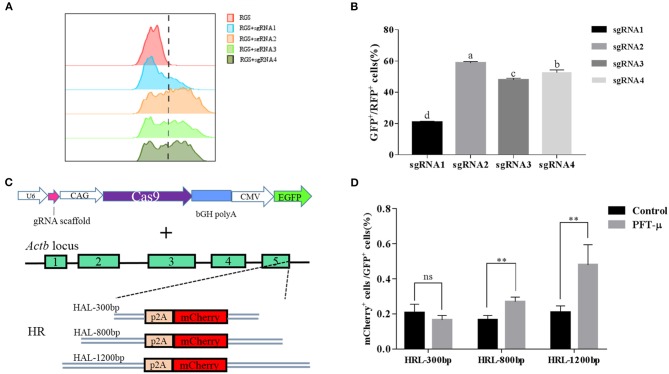
**(A)** The RGS reporter system was used to determine the efficiencies of sgRNAs; the cleavage activities were evaluated by FACS and are presented as the ratio of GFP^+^/RFP^+^ cells to all transfected cells; **(B)** Histograms show the relative cleavage activity of different sgRNAs; **(C)** Schematic overview of the strategy used to target the *Actb* locus in buffalo fetal fibroblast cells; **(D)** Knock-in efficiencies were evaluated by FACS and are presented as the ratio of mCherry^+^ cells to all transfected cells. **P < 0.01, ns, no significant difference.

**Figure 4:**
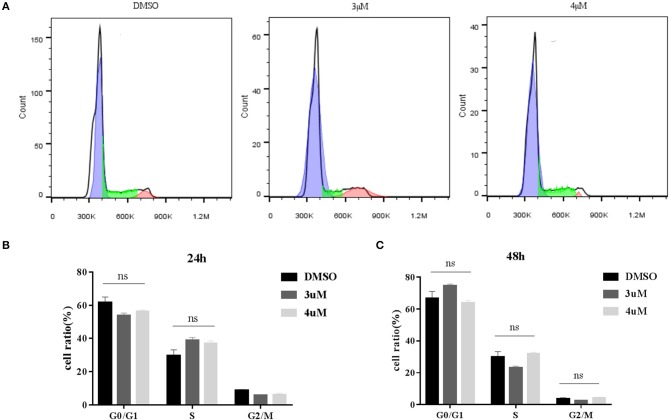
**(A)** Cell cycle of BFF cells with different concentration of PFT-μ; **(B)** Histograms show the change of cell cycle in BFF cells with different concentration of PFT-μ for 24 h; **(C)** Histograms show the change of cell cycle in BFF cells with different concentration of PFT-μ for 48 h.

We next determined whether addition of PFT-μ to the culture medium affects the knock-in efficiency when using the HR-based method. We constructed three donor plasmids with HA lengths of 300, 800, and 1,200 bp. To assess the knock-in efficiencies, we aimed to fuse a *p2A-mCherry* reporter gene to the last codon of the β-*actin* gene in BFF cells. We designed four sgRNAs targeting the defined locus (Table S2), and the results from the RGS reporter system showed that sgRNA2 was the most efficient; therefore, this sgRNA was selected for subsequent flow cytometry experiments (Figures 3A,B). A schematic overview of the strategy used to target the *Actb* locus in BFF cells is shown in Figure 3C. Seven days after transfection, the knock-in efficiencies for HAs of different lengths in the presence of PFT-μ were evaluated and are presented as the percentage of cells expressing mCherry, as determined by flow cytometry (Figure 3D). Higher knock in efficiencies in BFF cells were observed with an HA length of 1,200 bp, and PFT-μ significantly improved the HR efficiency when the HA length was 800 or 1200 bp (Figure 3D). Cells cycle were analyzed after incubated with different concentrations of PFT-μ for 24 or 48 h, the results indicate that no significant differences in cell cycle with the presence of PFT-μ or not (Figure 4).

### Establishment of Y-Chr-eGFP Transgenic Cells

**Figure 5:**
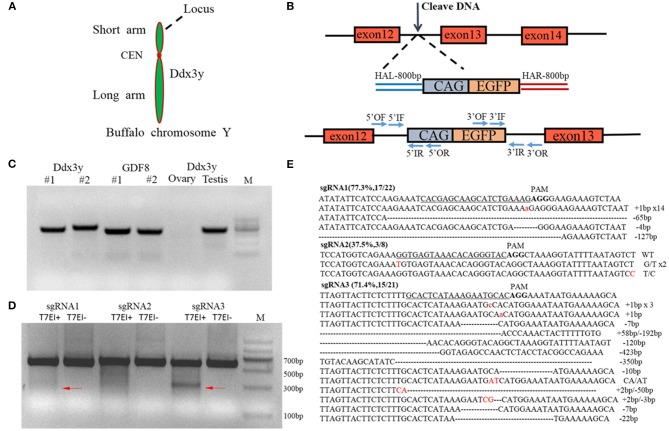
**(A)** The targeted gene on the Y chromosome, *Ddx3y*, is located on the long arm; **(B)** Schematic overview of the strategy used to target the *Ddx3y* locus. HAL/HAR, left/right homology arm; **(C)** Identification of the sex of isolated buffalo fetal fibroblast cells. The *GDF8* gene was used as a control; **(D)** T7EI assay of three different sgRNAs; **(E)** The sequencing results for a single clone.

**Figure 6:**
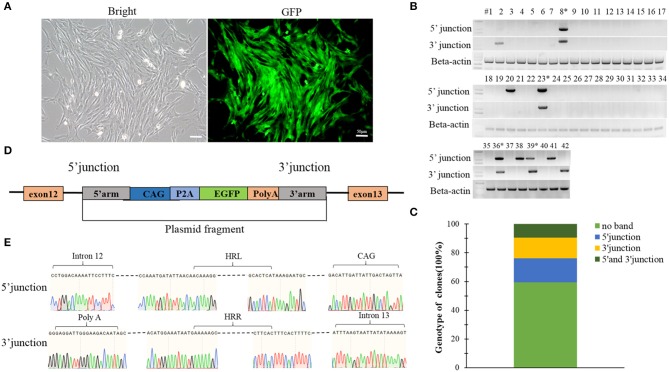
**(A)** A single puromycin resistant colony cultured for 7 days after withdrawing puromycin; **(B)** Screening for the integrated transgene in all 42 selected colonies; **(C)** The percentage of the 42 colonies with a given genotype (no integration, only 5′ junction, only 3′ junction or both 5′ and 3′ junctions); **(D)** Schematic overview of the strategy used to target the *Ddx3y* locus; **(E)** Sequences of PCR products amplified from the 5′ and 3′ junctions. Scale bar = 50 μm.

A construct was designed to integrate the *eGFP* gene into intron 12 of the *Ddx3y* gene (Gene ID: 783057) located on Y chromosome in BFFs as shown in Figures 5A,B. The sex of harvested BFF cells was determined by amplifying *Ddx3y*, and the *GDF8* gene located on an autosome was amplified as a control. The results of sex identification showed that male BFFs were successfully obtained (Figure 5C). Three sgRNAs were designed to target the intron of the *Ddx3y* gene (Table S1), and based on the combined results of T7EI (Figure 5D) and sequencing (Figure 5E), sgRNA3 was chosen for further experiments. Px459 and an HR donor carrying a CAG promoter, an *eGFP* gene, and 5′ and 3′ homologous sequences were co-transfected to BFFs. A schematic overview of the strategy used to target the *Ddx3y* locus is shown in Figure 5B. Screening for Y-Chr-eGFP transgenic cells was then performed. Only a few cells expressing the puromycin gene survived in the presence of puromycin for 4 days. After puromycin was removed and cells were cultured for 7–10 days, individual eGFP-positive cell colonies were recovered (Figure 6A). We randomly picked 42 puromycin-resistant eGFP-positive cell colonies for proliferation, and genotyped the expanded cell colonies using PCR and DNA sequencing to confirm that the desired gene integration had occurred (Figure 6B). The desired integration was only found in 9.2% of the colonies (Figure 6C). The results of PCR and DNA sequencing of these colonies also confirmed that fragments of the expected sizes, 998 bp for the 5′ junction and 1,101 bp for the 3′ junction, were amplified, indicating that the cells contained the correctly integrated *eGFP* gene (Figures 6D,E).

### Comparison of the Relative Cell Viability and Proliferation Rate of Y-Chr-eGFP Transgenic and Non-transgenic Cells

**Figure 7:**
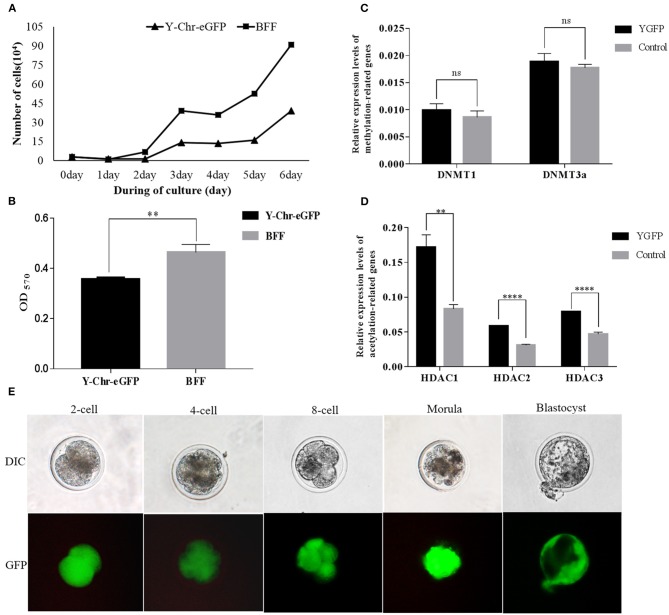
**(A)** Proliferation of Y-Chr-eGFP transgenic and non-transgenic buffalo fetal fibroblast cells grown for 144 h in a 6-cell dish; **(B)** Cell viability of Y-Chr-eGFP transgenic and non-transgenic buffalo fetal fibroblast cells evaluated by MTT assay; **(C)** Relative expression levels of DNA methylation-related genes in Y-Chr-eGFP transgenic and non-transgenic fetal fibroblast cells determined by real-time PCR. **(D)** Relative expression levels of histone acetylation-related genes in Y-Chr-eGFP transgenic and non-transgenic fetal fibroblast cells determined by real-time PCR. Values are reported as the mean ± SEM; **P < 0.01, ****P < 0.0001. **(E)** Y-Chr-eGFP transgenic embryos derived from SCNT at different stages of development.

The proliferation rate of Y-Chr-eGFP transgenic cells was found to be significantly lower than that of non-transgenic cells (Figure 7A). Also, the relative cell viability determined by MTT assay was significantly lower for Y-Chr-eGFP transgenic cells compared with non-transgenic cells (Figure 7B).

### Analysis of Epigenetic-Related Gene Expression in Y-Chr-eGFP Transgenic and Non-transgenic Cells

Comparison of the relative gene expression levels of methylation-related genes in the Y-Chr-eGFP transgenic and non-transgenic cells revealed that the expression levels of *DNMT1* and *DNMT3a* were similar between the two groups (Figure 7C). However, the expression levels of the acetylation-related genes, *HDAC1, HDAC2*, and *HDAC3*, were significantly higher in Y-Chr-eGFP transgenic cells than in non-transgenic cells (Figure 7D).

### Developmental Competence of Y-Chr-eGFP Transgenic and Non-transgenic Embryos

Cloned Y-Chr-eGFP buffalo embryos produced using donor cells with well-defined genotypes, showed strong expression of the eGFP gene during embryo development *in vitro* (Figure 7E). This demonstrates that Y-Chr-eGFP transgenic fibroblasts used as donor cells could be successfully reprogrammed and form into blastocysts *in vitro*. The rates of development of cloned embryos produced from Y-Chr-eGFP transgenic and non-transgenic cells are in presented in Table 1. The cleavage rate was significantly higher (*P* < 0.05) for embryos produced from non-transgenic cells than for those derived from Y-Chr-eGFP transgenic cells.

**Table 1:** Developmental competence of cloned embryos produced from transgenic and non-transgenic fetal fibroblast cells.

**Donor cell type**	**Reconstructed embryos (*n*)**	**Cleaved embryos *n* (%)**	**Blastocysts *n* (%)**
Fetal fibroblast cells	199	71 (36.01+0.42)^a^	9 (13.55+1.11)^a^
Y-Chr-eGFP transgenic fetal fibroblast cells	242	76 (30.94+1.56)^b^	10 (13.45+0.84)^a^

*Blastocyst rate data are from 5 trials. Values are mean ± SEM. Values with different superscript within the same column differ significantly (p < 0.05)*.

## Discussion

Genetically modified animals play a remarkable role in agriculture and biomedicine (69–72). Limitations in the efficiency of genetic modification of primary mammalian cells including fibroblasts greatly limits the production of transgenic animals. Many attempts have been made to optimize the efficiency of integration of exogenous DNA into genomes (73–75). HR is one of the most important method to conduct precise gene editing (73), which is generally inefficient in mammalian cells for that HR pathway functions during the late S/G2 phase (76, 77). However, DSBs can stimulate the HR pathway (76) and HR requires the presence of homologous sequences (78, 79), using CRISPR/Cas9 in combination with a template DNA sequence can greatly increase the rate of HR. The HA length of donor template significantly changed the integration efficiency in BFF cells, which is consistent with previous studies (77, 80).

PFT-μ is a small molecule that inhibits p53 binding to mitochondria by reducing its affinity to the anti-apoptotic proteins Bcl-xL and Bcl-2 but has no effect on p53-dependent trans-activation (61). In this study, we found that addition of PFT-μ to the medium can significantly improve the efficiency of precise genome editing in BFF cells, which indicated that p53 may be one of the reasons for the decreased HDR efficiency in primary cells. PFT-μ can induce cell cycle arrest in acute leukemia cell lines (81), in addition HR is active only during the late S/G2 phase (82, 83). PFT-μ probably improve the knock-in efficiency of BFF cells by decreasing the apoptosis of cells with DSB, not by changing the cell cycle. Considering of the severe chromatin damage brought by p53 inhibition (84, 85), a short-term use of p53 inhibitors not only provide a window for genome editing temporarily, but also the expression level of p53 can restoration (59).

The selection of positive cells using drugs is a very crucial method for obtaining transgenic cells to produce transgenic animals (68, 86–88). Although the efficiency at which insertion/deletions are introduced by the CRISPR/Cas9 system is very high (54, 89), the knock-in efficiency still cannot satisfy the demands of agricultural production, which significantly limits the application of this technique (55). As has been indicated in many reports, drug selection can irreversibly harm the cells, the cells surviving drug screening had a decreased survival rate after multiple cell passages (55, 88).

DNA methylation and histone acetylation are important epigenetic events, the variations in the expression profile of epigenetic related genes in embryos and cloned animals are suggested to be linked with the reprogramming process (90–92). Some reports showed that the epigenetic modification of donor cells can affect the efficiency of reprogramming in SCNT (36, 37, 40). A previous research has shown that increased histone acetylation is associated with more effective formation of DNA replication complexes and can facilitate cell proliferation (93). In our study, no significant difference (*P* < 0.05) was observed in the transcript level of *DNMT1* and *DNMT3a*, and the expression level of *HDAC1, HDAC2, HDAC3*, which was found to be higher in Y-Chr-eGFP transgenic than in non-transgenic cells. The decreased histone acetylation levels may be one of the reasons for the lowered cell proliferative potential of transgenic cells and lowered cleavage rate of clone embryos from Y-Chr-eGFP transgenic BFF cells. Our results are consistent with Beyhan et al. who reported that the expression levels of HDACs in donor cells are related to the potential of embryos to develop to different embryonic stages (91). However, some research indicated the agent 5-aza-dC used for demethylation of DNA couldn't improve the development potential of cloned embryos (43, 44, 46), which indicated that the relationship between the level of DNA methylation status of donor cells and the developmental efficiency of cloned embryos needs to be further elucidated.

In conclusion, our study demonstrates that a Y chromosome tracer can be realized by CRISPR/Cas9 mediated HDR, p53 may be one of the reasons for the decreased HDR efficiency in primary cells. The raised expression level of *HDACs* in Y-Chr-eGFP transgenic BFFs could be correlated with reduced cell proliferative potential and developmental potential of transgenic cloned embryos. As far as we know, this is the first report of applying CRISPR/Cas9-mediated gene editing to solve the challenge of controlling the sex ratio of domestic animals. This work provides a rapid and non-invasive method to identify the sex of mammalian implantation embryos and lays a solid foundation for the generation of Y-Chr-eGFP transgenic buffalo and other transgenic species.

## Data Availability Statement

All datasets generated for this study are included in the article/Supplementary Material.

## Ethics Statement

The animal study was reviewed and approved by Animal Care & Welfare Committee of Guangxi University (GXU2016-013).

## Author Contributions

MZ, XZ, and JN conceived the study. XZ, JN, YT, WH, KX, and CP performed the experiments. XZ, JN, XL, YL, and MZ analyzed the data and wrote the manuscript. All authors reviewed the manuscript and approved the final version of the paper.

## Conflict of Interest

The authors declare that the research was conducted in the absence of any commercial or financial relationships that could be construed as a potential conflict of interest.
