# Supplementary material for: Generation of Transgenic Cloned Buffalo Embryos Harboring the EGFP Gene in the Y Chromosome Using CRISPR/Cas9-Mediated Targeted Integration
Source: Front Vet Sci. 2020 Apr 23;7:199. doi: 10.3389/fvets.2020.00199 (PMC7212351; doi:10.3389/fvets.2020.00199)
Supplement: Supplementary file 1 [file Data_Sheet_1.docx]

**Supplementary information**

Generation of Transgenic Cloned Buffalo Embryos Harboring the EGFP Gene in the Y Chromosome Using CRISPR/Cas9-Mediated Targeted Integration

Running title: Optimization of Homologous Recombination in Transgenic Cloned Buffalo Embryos

Xiuling Zhao^1,*^, Junyu Nie^1,*^, Yuyan Tang^1^, Wengtan He^1^, Kai Xiao^1^, Chunying Pang^2^, Xianwei Liang^2^, Yangqing Lu^1,**^, Ming Zhang^1, **^

^1^ State Key Laboratory for Conservation and Utilization of Subtropical Agro-Bioresources, Animal Reproduction Institute, Guangxi University, Nanning, 530004, Guangxi, PR China

^2^ Key Laboratory of Buffalo Genetics, Breeding and Reproduction Technology, Ministry of Agriculture and Buffalo Research Institute, Chinese Academy of Agricultural Science, Nanning, Guangxi 530001, PR China

* These two authors contributed equally to this work.

** Corresponding author. E-mail address: luyangqing@126.com (Y.Q. Lu), mingzhang@gxu.edu.cn (M. Zhang)

**Contents:**

**Figure S1.** Map of the RGS plasmid.

**Figure S2.** The percentage of transfected cells, as determined by mCherry expression, was affected by voltage.

**Figure S3.** The percentage of transfected cells, as determined by mCherry expression, was affected by the number of pulses.

**Figure S4.** In vitro development of non-transgenic cloned embryos from buffalo.

**Table S1.** The sequences of the sgRNAs targeting the Ddx3y gene from buffalo.

**Table S2.** The sequences of the sgRNAs targeting the Actb gene from buffalo.

**Table S3.** Primers used for amplification of 5’ and 3’ junctions.

**Table S4.** Primers used for amplification of epigenetic-related genes.


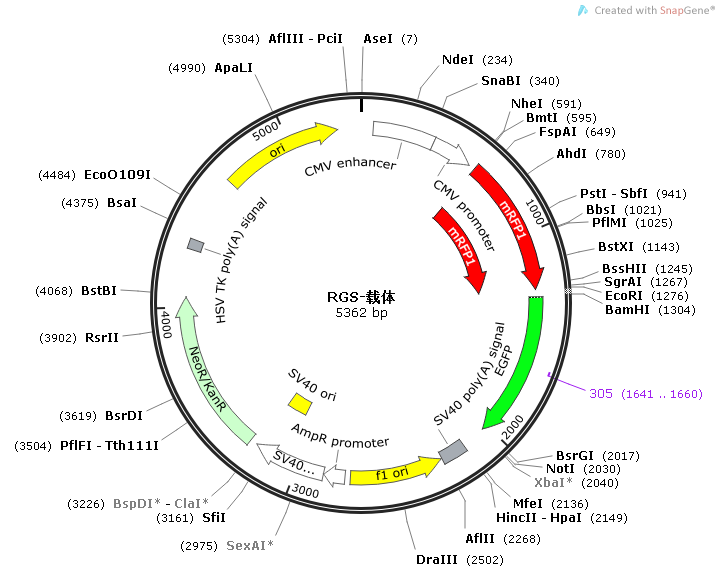


**Figure S1.** Map of the RGS plasmid.


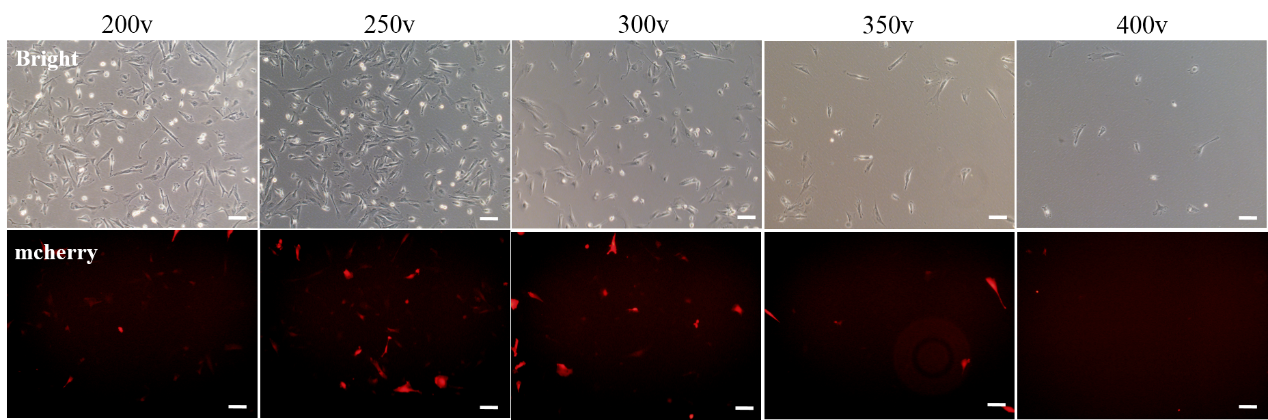


**Figure S2.** The percentage of transfected cells, as determined by mCherry expression, was affected by voltage.
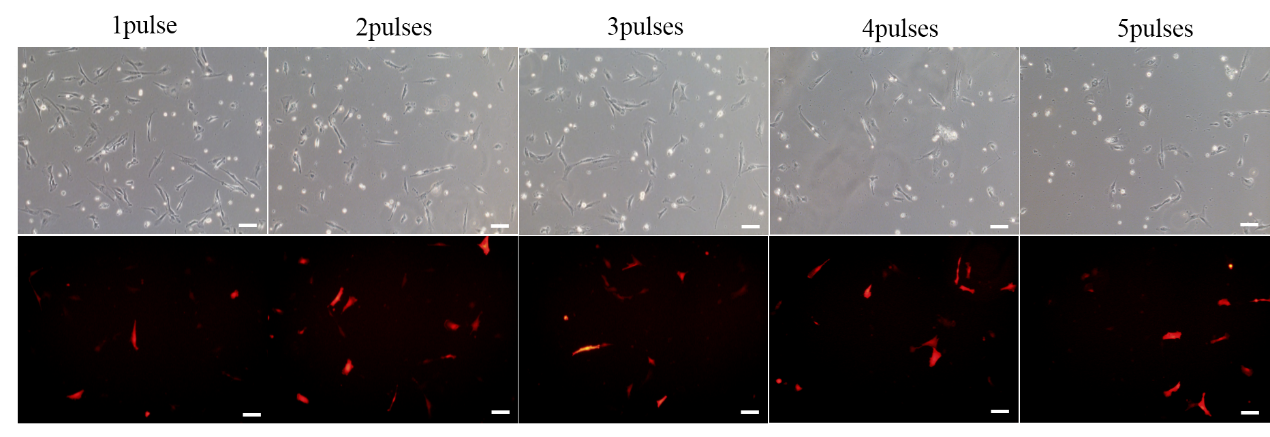


**Figure S3.** The percentage of transfected cells, as determined by mCherry expression, was affected by the number of pulses.


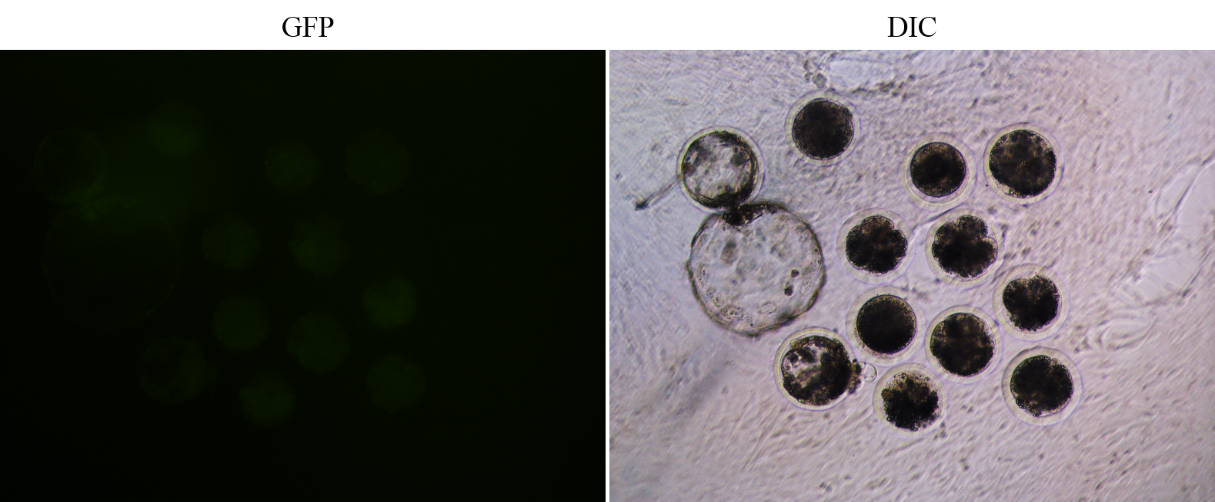


**Figure S4.** In vitro development of non-transgenic cloned embryos from buffalo.

| sgRNA | Sequence (5’-3’) |
| --- | --- |
| sgRNA1 | CACGAGCAAGCATCTGAAAG |
| sgRNA2 | GGTGAGTAAACACAGGGTAC |
| sgRNA3 | GCACTCATAAAGAATGCACA |

**Table S1.** The sequences of the sgRNAs targeting the Ddx3y gene from buffalo

| sgRNA | Sequence (5’-3’) |
| --- | --- |
| sgRNA1 | GATCCACATCTGCTGGAAGG |
| sgRNA2 | CGTCCACCGCAAATGCTTCT |
| sgRNA3 | GCAGATGTGGATCAGCAAGC |
| sgRNA4 | CCACCGCAAATGCTTCTAGG |

**Table S2.** The sequences of the sgRNAs targeting the Actin gene from buffalo

| Primer | Sequence (5’-3’) |
| --- | --- |
| 5’ OF | TCAACTCATTTTCATGTGGC |
| 5’ OR | GGCTATGAACTAATGACCC |
| 5’ IF | GTACAGTAGTAATGGCAGTG |
| 5’ IR | GGCTATGAACTAATGACCC |
| 3’ OF | CCCACTGTCCTTTCCTAA |
| 3’ OR | GGTGGTGCTGATATTGGT |
| 3’ IF | CCCACTGTCCTTTCCTAA |
| 3’ IR | GGATGTCACTTGTTGGTG |

**Table S3.** Primers used for amplification of 5’ and 3’ junctions

| Gene | Primer | Sequence (5’-3’) | Product size |
| --- | --- | --- | --- |
| DNMT1 | F | CTCAGAAGGGAGACGTGGAG | 138 bp |
|  | R | TAGTAGTCACAGTAGCTGAGGA |  |
| DNMT3a | F | GTGCTGTCTCTATTCGATGG | 188 bp |
|  | R | CCATTCCTGGATATGCTTCTG |  |
| HDAC1 | F | ATCGGTTAGGTTGCTTCAATCTG | 168 bp |
|  | R | TTGTATGGAAGCTCATTAGGGA |  |
| HDAC2 | F | ACAGGGTCATCCCATGAAAC | 115 bp |
|  | R | TTCTTCAGCAGTGGCTTTAT |  |
| HDAC3 | F | ATCCGGATGGAGCGTGAAGT | 137 bp |
|  | R | GTGGCTACACTGTCCGGAAT |  |
| GAPDH | F | TCAAGAAGGTGGTGAAGC | 122 bp |
|  | R | CCCAGCATCGAAGGTAGA |  |

**Table S4.** Primers used for amplification of epigenetic-related genes
